# High-Pressure-Assisted Large-Area (>2400 mm^2^) Sintered-Silver Substrate Bonding for SiC Power Module Packaging

**DOI:** 10.3390/ma17081911

**Published:** 2024-04-20

**Authors:** Jiafeng Xue, Xin Li

**Affiliations:** 1 School of Material Science and Engineering, Tianjin University, Tianjin 300072, China; xue_jiafeng@163.com; 2 Tianjin University Binhai Industrial Research Institute Co., Ltd., Tianjin 300072, China

**Keywords:** sintered silver, high pressure assisted, large area, substrate bonding, reliability

## Abstract

The emergence of new semiconductor devices puts forward higher requirements for packaging technology. Sintered silver technology has gradually developed into critical packaging technology in silicon carbide power module packaging due to its good heat dissipation performance and reliability. However, high sintering drive requirements, low sintering densification, and high thermal–mechanical stresses limit the application of sintered silver technology for large-area bonding. In this study, the high-pressure-assisted (≥10 MPa) large-area sintered-silver interconnection process between a substrate and baseplate was discussed. C-scan acoustic microscopy, warpage testing, and microanalysis were used to analyze the effects of drying methods, sintering pressure, and holding time on the sintered joints, and thermal fatigue reliability tests were conducted on large-area sintered silver joints. The results demonstrated that the quality of large-area sintered joints obtained via open-face convective drying is higher than that via close-face convective drying. Combining the performance of sintered joints and productivity, the recommended process condition is determined as follows: open-face convective drying, sintering temperature of 250 °C, sintering pressure of 15 MPa, and holding time of 5 min. Large-area sintered joints have outstanding reliability, with slight delamination of the sintered layer at the corners and no cracking after 1000 cycles of temperature cycling.

## 1. Introduction

In recent years, the rapid development of the new energy vehicle (NEV) industry has promoted the application and innovation of power electronic devices for electrical power conversion. The performance limitations of traditional silicon-based devices are restricting the application of power modules in the new energy vehicle industry. Due to excellent material properties, silicon carbide (SiC) power modules have been suggested as the most attractive substitute for silicon-based power modules [1]. As the essential wide-bandgap (WBG) semiconductor device, SiC devices have superior electrical and physical properties, as well as high junction and operating temperatures. The significant increase in junction and operating temperatures can help expand the power device’s high-temperature application capability [2,3,4]. The emergence of SiC devices has raised new challenges to packaging technology in meeting the requirements of devices in terms of performance, efficiency, and reliability [5].

High-quality interconnections of die bonding and substrate bonding are critical in the packaging technology of SiC power modules [6]. Thermal interface materials used for die bonding and substrate bonding have a significant impact on the heat dissipation and reliability of the module. Currently, the interconnection materials used for power modules in new energy vehicles are primarily tin-based solder alloys, which cannot meet the thermal conductivity (490 W/m·K), operating temperature (>200 °C), and reliability requirements of SiC devices due to their low thermal conductivity (59 W/m·K) and operating temperatures (<150 °C), as well as creep-fatigue concerns [7,8]. Alternative thermal interface materials and technologies need to be developed to meet the performance and reliability of SiC devices.

Compared to conventional solder alloys, sintered silver has high thermal conductivity (240 W/m·K) and a high melting point (961 °C), which means that it possesses better heat dissipation properties and operating temperatures that meet the heat dissipation and high-temperature resistance requirements of SiC devices [6]. In addition, conventional solder is prone to fatigue cracking when undergoing conditions such as temperature cycling or high-frequency vibration. In contrast, sintered silver joints have better lifetime and reliability [9,10,11]. Currently, there are more studies on using sintered silver technology for die bonding in SiC devices. Li et al. [12] attached 3 × 3 mm SiC dies with thick Ti\W\Au metallization on aluminum nitride (AlN)-based directly bonded copper substrates using the sintering of Ag nanoparticle paste. Chen et al. [13] obtained strong joints with a shear strength of > 500 kgf and steady-state thermal resistance of 0.273 °C/W under ultra-low temperature (180 °C) and low-pressure-assisted (≤5 MPa) conditions. Michele Calabretta et al. [8] studied silver sintering for an attached SiC die under different pressures (with pressure values between 10 MPa and 30 MPa). In addition to pressure-free and pressure-assisted sintering methods, some new ones have been proposed, such as electric current-assisted sintering (ECAS) and selective laser sintering [14,15,16].

In comparison to the die bonding area (<100 mm^2^), the substrate bonding area in SiC devices can reach 2000 mm^2^, presenting a significant challenge for sintered silver technology, including high requirements for the sintering driving force, insufficient sintering densification, and high thermal–mechanical stresses that cause cracks and the warpage of sintered joints. Sintering pressure, sintering time, and sintering temperature are critical parameters for high-quality sintered joints, and a decrease in one of the three can be adjusted for by an increase in the other two. Tan et al. [17] obtained sintered silver joints with an area of 800 mm^2^ under low pressure (≤3 MPa) via double printing, and Zhang et al. [18] obtained sintered silver joints with an area of 625 mm^2^ under low pressure (≤2 MPa) using the single-printing method. Whether it is the double-printing or single-printing method combined with segmental heating, the preparation process was complicated, and the low sintering pressure needed to be combined with the higher sintering temperature (280 °C) and longer sintering time (>2 h), which reduced productivity and was not conducive to large-scale applications. Increasing the sintering pressure decreases sintering temperatures and holding times, reducing thermo-mechanical stresses in the package component and increasing production efficiency [19]. At the same time, the high sintering pressure increases the sintering drive force and densification of sintered layers. Consequently, high assisted pressure is critical for large-area substrate bonding, and it is significant for studying the high-pressure-assisted sintering process to realize reliable substrate bonding in SiC power modules.

In this study, the high-pressure-assisted (≥10 MPa) sintering process was used for large-area substrate bonding (>2400 mm^2^). The effects of different drying methods on sintered joint properties were studied first, followed by a quantitative study of the impact of different sintering pressures and holding times on the bonding quality of the sintered joint. Finally, large-area sintered joints were tested for temperature cycling.

## 2. Materials and Methods

### 2.1. Constituents of Silver Paste

**Figure 1:**
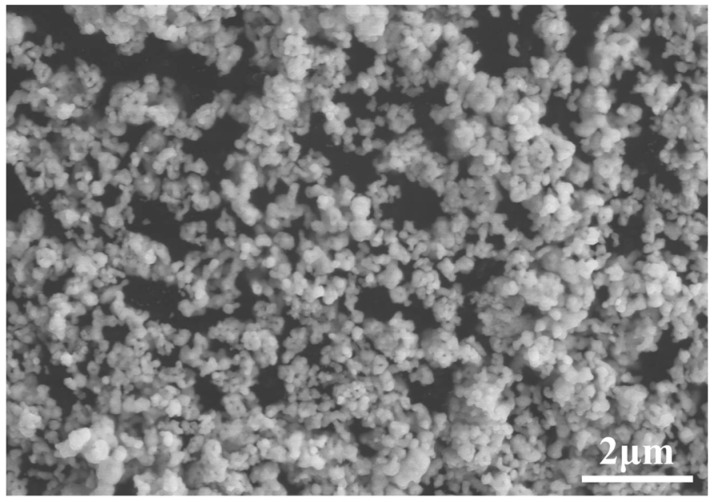
SEM observation of silver particles in the nano-silver paste.

A commercially available nano-silver paste made by MacDermid Alpha (Waterbury, CT, USA) was used in this experiment. The microstructure of the paste was characterized via scanning electron microscopy (SEM), as shown in Figure 1. The silver paste contains spherical nanoparticles with diameters less than 100 nm. In addition to nano-silver particles, solvents such as dispersants, binders, and thinners are added to silver paste. The dispersants cover the surface of the silver nanoparticles and prevent self-sintering caused by the surface free energy. The binders promote bonding and prevent the cracking of the silver paste during preheating. The thinners regulate the viscosity and fluidity of the silver pastes to ensure that it is suitable for stencil printing [20].

**Figure 2:**
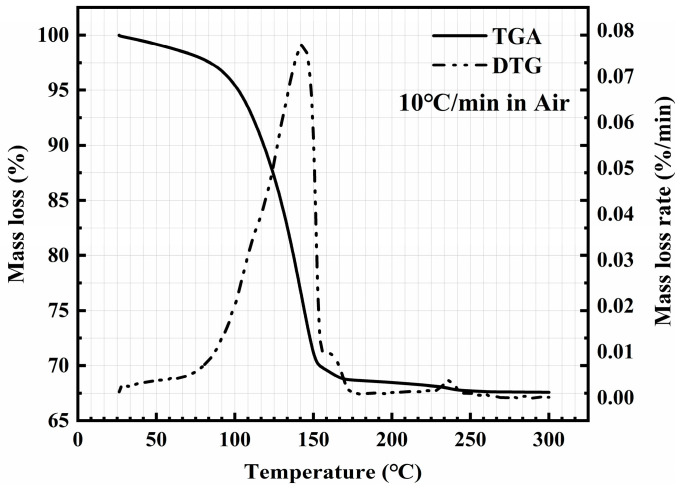
Thermalgravimetric analysis and derivative thermogravimetry of the silver paste.

The volatilization process of the solvent of silver pastes with temperature change in the air was analyzed via thermogravimetric analysis (TGA) and derivative thermogravimetry (DTG), and the temperature was increased from 25 °C to 300 °C with a heating rate of 10 °C/min. As shown in Figure 2, the silver content of the silver paste is about 67.5 wt%, and the maximum mass loss rate occurs at 140 °C.

### 2.2. Sample Preparation

**Figure 3:**
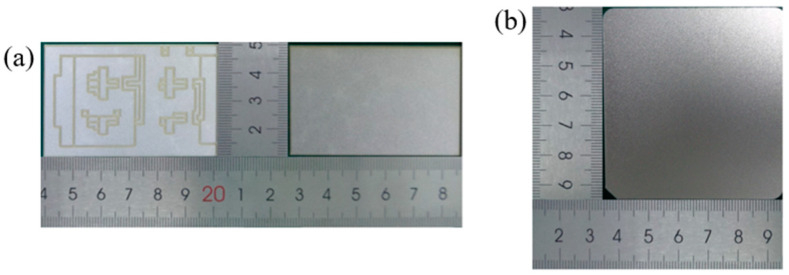
Schematic of the substrate and baseplate: (**a**) DBC substrate; (**b**) copper baseplate.

Large-area sintered samples were fabricated by bonding direct bond copper (DBC) substrates and copper baseplates. As shown in Figure 3, the DBC substrate is 40 × 62 mm in size, with the thickness of the copper layer and sandwich alumina being 300 and 635 µm, and the top copper layer is etched. The copper baseplate is 60 × 65 mm in size with a thickness of 3 mm. To form higher-quality joints and prevent oxidation, the whole surface of the substrate is silver-plated, with a thickness of 0.5–0.6 μm, and the copper baseplate surface is silver-plated, with a thickness of 3 μm. The silver paste was printed with a 300 μm thickness on the surface of the copper baseplate. The printing parameters mainly include a squeegee pressure of 30 N, a printing speed of 30 mm/s, and a release speed of 10 mm/s. To ensure the consistency of the printed dimensions, a customized stencil is used to control the thickness of the paste, and a jig is used to calibrate the position.

**Figure 4:**
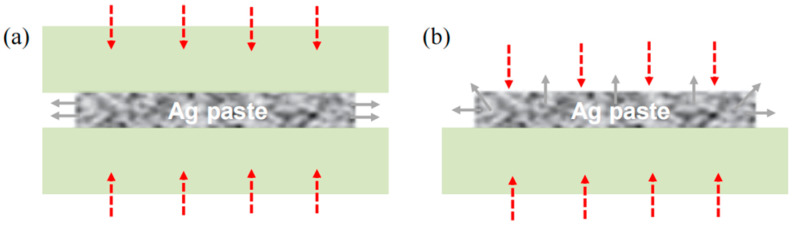
Schematic diagram of CVectDry and OVectDry: (**a**) CVectDry; (**b**) OVectDry. Red arrows represent the direction of heat imposition, and gray arrows represent the venting direction of the solvent.

After printing, the drying step was carried out to remove the solvents from the silver paste. Appropriately drying methods of wet-printed Ag paste are vital to the bonding quality of sintered joints. It is important to ensure that the paste is sufficiently dry, and incomplete drying will result in outgassing during sintering, causing delamination, voids, and less densification. As shown in Figure 4, the two most commonly used convective drying methods are close-face convective drying (CVectDry) and open-face convective drying (OVectDry). CVectDry (Figure 4a) relies on convection between the object overlaid on the wet Ag paste and the surrounding gas to transfer heat into the paste and dry it. The advantage of this method is that the surface of the connected object can make initial contact with the wet silver paste, resulting in better wetting adhesion. The disadvantages of this method include the difficulty in maintaining the original print size and shape, and the solvent can only laterally exit or vent. To minimize this effect, a small printing area and short lateral solvent venting distance are recommended. OVectDry (Figure 4b) also relies on the surrounding warm gas, but heat can be convectively transferred directly to the surface of the wet paste, and the solvent can vent vertically from the exposed surface. The heating method of the OVectDry is similar to baking a cake in an oven. As a result, OVectDry can remove solvents from the wet paste more quickly, and it can be applied to larger printing areas than CVectDry. The disadvantage of OVectDry is that no initial contact is made between the silver paste and the connected object, and the dry contact surface causes poor wetting adhesion [21].

**Figure 5:**
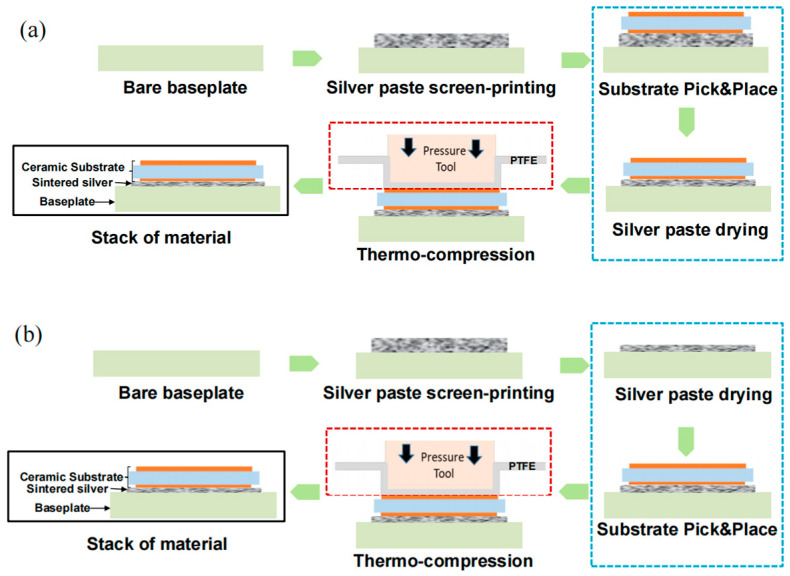
Schematic of the sintering process flow for different drying methods: (**a**) CVectDry; (**b**) OVectDry.

Figure 5 shows the process flow for the two drying methods used in this experiment. The information in the blue border of Figure 5 indicates the different process flow between CVectDry and OVectDry, with the difference being the inconsistent order of substrate placement and silver paste drying. In the CVectDry method, after printing the silver paste on the baseplate, the substrate is directly attached to the printed silver paste and then put into the oven for drying. The printed paste is still wet when the substrate is attached and easily forms the initial surface contact. However, in large-area bonding, since the solvent can only laterally exit or vent, it may take a long time to remove the solvent, resulting in the incomplete escape of the solvent. In contrast, OVectDry removes the solvent sufficiently by drying the printed paste on the baseplate and then attaching the substrate. The solvent can be easily removed using this method. However, it can be challenging to create a tight surface contact between the dried silver paste and the substrate, and the additional sintering drive may need to be applied.

**Figure 6:**
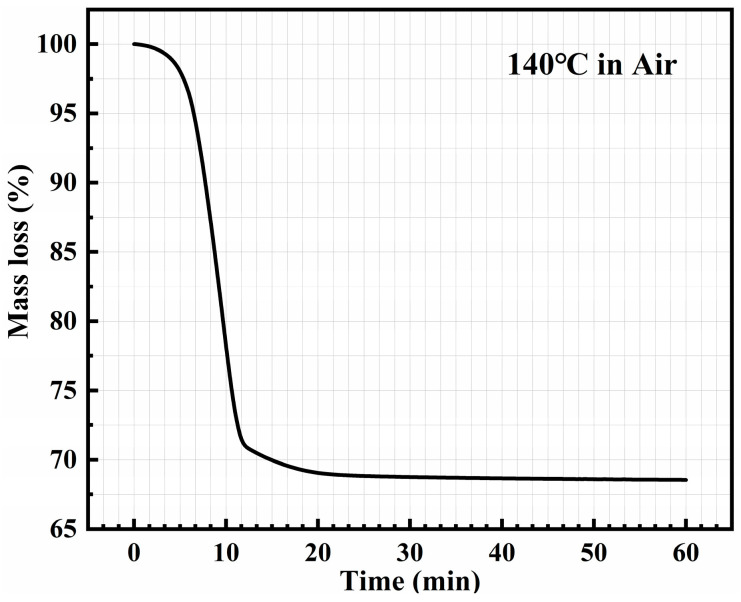
Thermalgravimetric analysis of the silver paste (140 °C in the air).

Based on the significance of the drying step, the first section of the research discussed the impact of different drying methods on joint performance. According to the results of TGA and DTG in Figure 2, the drying temperature was set at 140 °C. To determine the appropriate drying time, TGA was performed on the silver paste at 140 °C. As shown in Figure 6, the result of TGA showed that the solvent was almost completely removed when the drying time reached 20 min. Considering that the solvent is more difficult to remove when using CVectDry, the drying time was extended to 60 min to remove the solvent more sufficiently.

Table 1 lists the sintering process conditions of samples 1–2. Following drying, the samples were subjected to thermo-compression sintering with initial process parameters of 250 °C, 10 MPa pressure, and 5 min holding time. PTFE films (polytetrafluoroethylene) were used to keep the pressure consistent.

After determining the drying method for the silver paste, the sintering process was further explored by changing the sintering process parameters. The second section of the research discussed the impact of sintering pressure and holding time on joint performance. Table 2 lists the process exploration conditions of samples 3–8. The main equipment used in the sintering process flows includes printing equipment (EKRA serio 4000, EKRA Automatisierungssysteme GmbH, Bönnigheim, Germany), drying equipment (Binder M 115, BINDER GmbH, Tuttlingen, Germany), and sintering equipment (Boschman Sinterstar innovate-F-XL, Boschman, Duiven, The Netherlands). The atmosphere in the sintering process flows is air.

### 2.3. Characterization

Defects (e.g., cracks, voids, delamination) of the bonded interface can seriously affect the performance of sintered joints, such as heat dissipation and mechanical strength. As a nondestructive and quick detection method, C-scan acoustic microscopy (SAM 301, PVA TePla Analytical Systems GmbH, Westhausen, Germany) is used to observe defects such as voids and delamination at the interface of sintered silver layers. Based on the C-SAM images, the delamination area was calculated by Image J.

In the stack of material, ceramic, copper, and sintered silver have different coefficients of thermal expansion (CTE), and the mismatch of CTE will cause thermal stresses and warpage during the cooldown of sintered silver samples from the sintering temperature to room temperature. Excessive thermal stress and warpage can lead to crack initiation and propagation, influencing the performance of sintered silver joints. A 3D optical profilometer was used to scan the bottom surfaces of the copper baseplate for measurements of the warpage. Due to the 60 × 65 mm copper baseplate size, the warpage of long and short sides was tested separately.

Porosity quantifies the ratio of the volume of pores to the total volume of the sintered layer, and it is a physical parameter that is strictly related to sintering process conditions. Moreover, it affects the mechanical, thermal, and reliability performance of sintered joints [22,23]. In this study, SEM was used to observe the cross-section’s microstructure. Based on the SEM images, the porosity of the sintered layer was calculated via Image J software (ImageJ2).

Temperature cycling was used to evaluate the thermal fatigue reliability of large-area sintered silver joints. The temperature cycling profile of −55 °C to 125 °C was chosen based on Joint Electron Devices Engineering Council (JEDEC) standard number 22-A104D. The ramp rate was set at 10 °C/min, and a dwell time of 15 min at both temperature extremes was selected.

## 3. Results and Discussion

### 3.1. Performance Evaluation of Joints with Different Drying Methods

**Figure 7:**
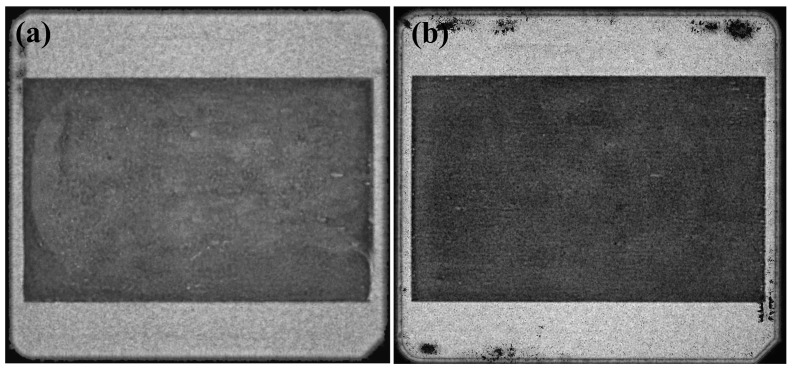
Nondestructive testing images of large-area sintering samples: (**a**) C-SAM images of SPE.1; (**b**) C-SAM images of SPE.2.

The C-SAM images of sintered samples 1–2 are shown in Figure 7. The images show that the sintered layer of sample 1 has slight voids and that the sintered layer is more homogeneous in the edge region than in the center region. In contrast, the homogeneous sintered layer is observed in sample 2. Considering the difference in the drying method of samples 1–2, the C-SAM results can be explained by the solvent removal process. When the drying method is CVectDry in large-area drying, the solvent can only vent or exit laterally, resulting in an inconsistent rate of solvent escape in the center and edge regions, which causes voids and inhomogeneity in the sintered layer. The homogeneous and defect-free sintered layer of sample 2 shows that the solvent was sufficiently removed after OVectDry.

**Figure 8:**
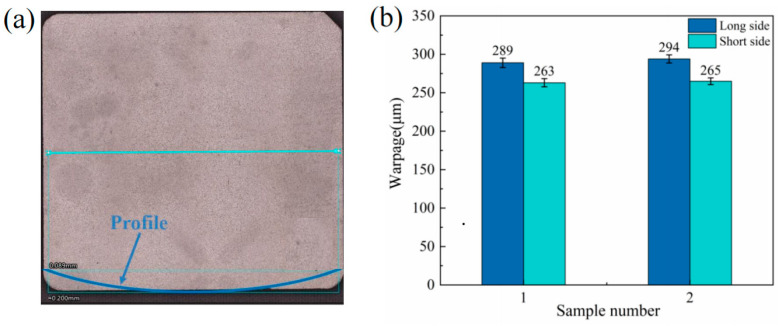
The warpage of the copper baseplate at room temperature: (**a**) the profile of the baseplate; (**b**) the warpage average of samples 1–2.

Ceramics, copper, and sintered silver have different coefficients of thermal expansion in the sintered interconnect structure. The high sintering pressure during the sintering process made samples difficult to deform. After sintering, the mismatch in coefficients of thermal expansion caused thermal stresses and warpage when the samples cooled from the sintering temperature to room temperature. As shown in Figure 8a, the 3D optical profilometer was used to measure the warpage of the copper baseplate at room temperature. Figure 8b shows the results of the warpage of samples 1–2. The difference in warpage is within 2%, which indicates that the different drying methods have almost no effect on the warpage of the copper baseplate.

**Figure 9:**
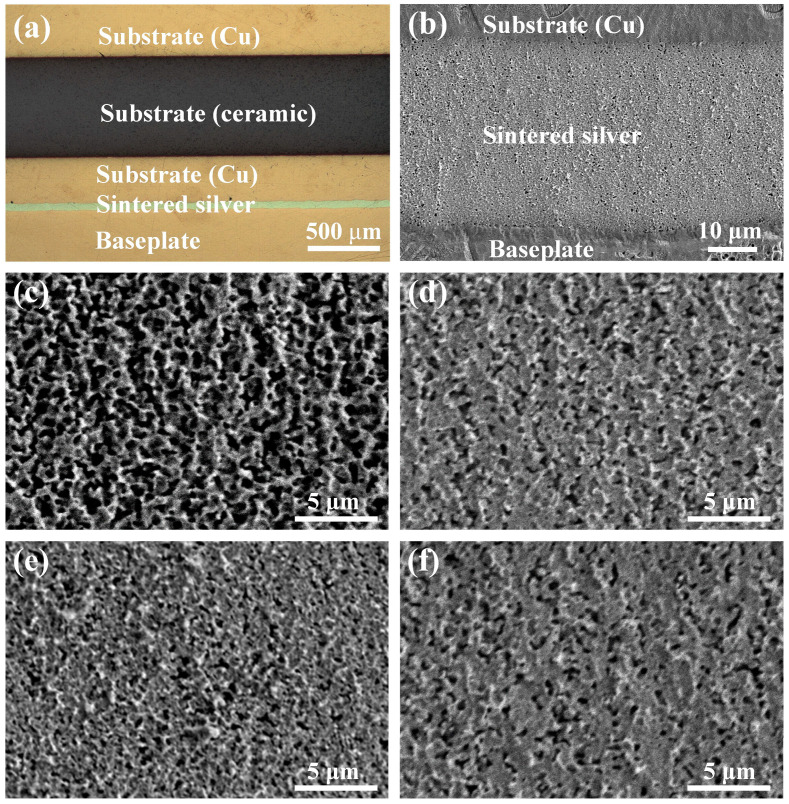
Cross-section images of the sintered joints: (**a**) optical micrograph of the joint; (**b**) SEM micrograph of the sintered layer; (**c**,**d**) central region of samples 1–2; (**e**,**f**) edge region of samples 1–2.

As shown in Figure 9a,b, the cross-sectional optical and SEM micrographs of the sintered joint indicate that the sintered silver layer is dense and there are no obvious defects in the sintered silver layer and the connection interface. The cross-section microstructure of the sintered layer’s center and edge regions is shown in Figure 9c–f. When CVectDry is used as the drying method, there is a significant difference in the porosity of the sintered layer between the center and edge regions. The porosity of the sintered layer is 37.1% in the center region and 18.64% in the edge region. In contrast, when OVectDry is used as the drying method, the porosity of the sintered layer is roughly 16% in both the center and the edge regions. Densification is the process of making the sintered layer denser. In general, as porosity decreases, the densification of the sintered layer increases. According to the measurement results of porosity, the sintered layer related to OVectDry is denser and more homogeneous, which is consistent with the C-SAM results. Residual solvents and the inconsistent rate of solvent escape cause excessive and nonuniform porosity in the sintered layer that is related to CVectDry, which can significantly affect the heat dissipation performance and reliability of sintered joints.

According to the above results, the sintered joint related to OVectDry has a higher quality than the joint related to CVectDry. When CVectDry is used as the drying method in this study, even though the substrate makes initial surface contact with the wet silver paste, the solvent is not adequately and uniformly removed, affecting the performance of the sintered joints. In contrast, OVectDry results in adequate solvent removal, and while initial surface contact is not formed, the additional sintering driving force provided by the assisted pressure of 10 MPa ensures a good sintered joint.

### 3.2. Performance Evaluation of Joints with Explored Process Parameters

**Figure 10:**
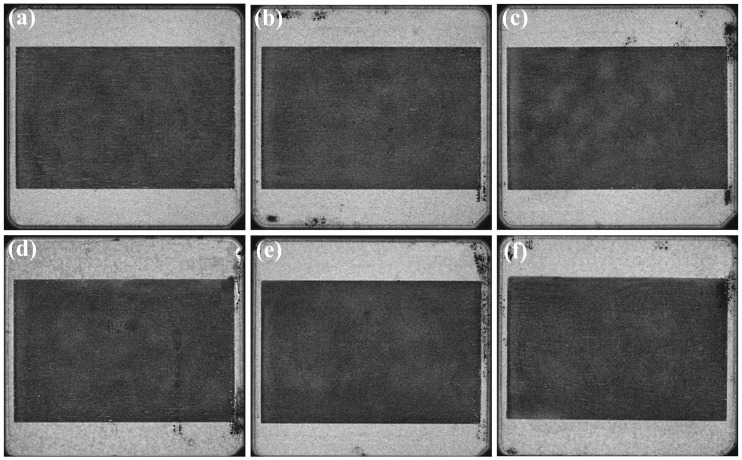
Nondestructive testing images of large-area sintering samples: (**a**–**f**) C-SAM images of samples 3–8.

**Figure 11:**
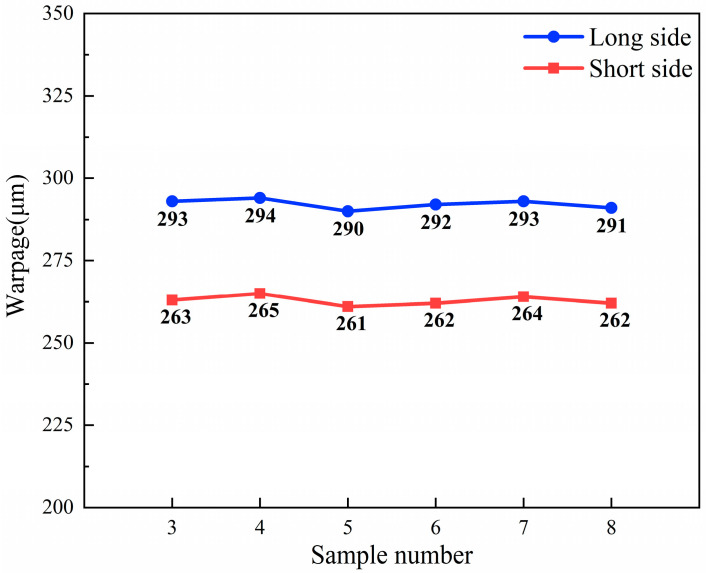
The warpage average of samples 3–8.

After determining the drying method as OVectDry, the effects of sintering pressure and holding time on the large-area sintered joints are studied while keeping other parameters constant. The C-SAM images of samples 3–8 are shown in Figure 10. Voids and delamination are not observed in the sintered layer for samples 3–8. The result demonstrates that when the sintering temperature is 250 °C, the sintered silver layers associated with sintering pressures between 10 and 15 MPa and holding times between 3 and 7 min are homogeneous and nearly defect-free. The warpage of samples 3–8 is shown in Figure 11. The difference in warpage is within 2%. The result demonstrates that the warpage of the copper baseplate is almost unimpacted by the sintering pressure and holding time.

**Figure 12:**
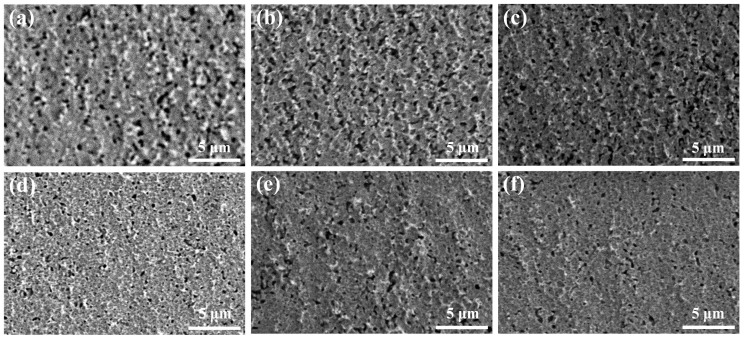
SEM images of the cross-section of the sintered layer: (**a**–**f**) Samples 3–8.

**Figure 13:**
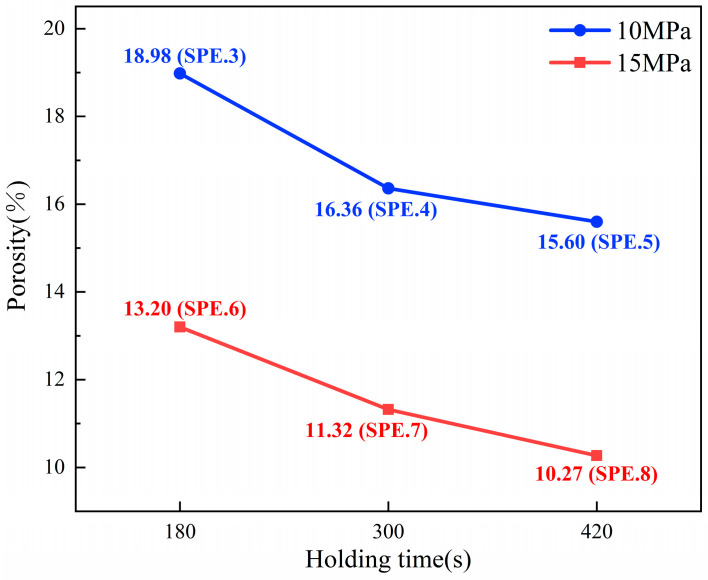
Porosity results of the sintered layers.

The microstructures of the cross-sections of the sintered layers of samples 3–8 were observed via SEM, as shown in Figure 12. The SEM images demonstrate that as holding time and sintering pressure increase, the densification of sintered layers increases. Figure 13 shows the porosity of the sintered layers calculated via Image J. The porosity results demonstrate that, for the same holding time, when the sintering pressure increased from 10 MPa to 15 MPa, the average porosity of the sintered layers decreased by 5.38%. This result is consistent with the Mackenzie–Shuttleworth equation [24]:

(1) $$\frac{d\rho }{dt}=\frac{3}{2}\left(\frac{\gamma }{r}+P_{applied}\right)\left(1-\rho \right)\left[1-\alpha {\left(\frac{1}{\rho }-1\right)}^{\frac{1}{3}}\ln\frac{1}{1-\rho }\right]\frac{1}{\eta }$$

In Equation (1) above, $\frac{d\rho }{dt}$ represents the rate of densification, r represents the radius of the particle, $\left(\frac{\gamma }{r}+P_{applied}\right)$ represents the driving force for densification, α represents the geometric constant, $P_{applied}$ represents the applied sintering pressure, ρ represents the density, γ represents the surface energy, and η represents the densification viscosity. When the surface energy of the silver particles is not sufficient in providing the driving force required for densification, the applied sintering pressure can provide additional sintering driving force. The rate of densification of the sintered joint can be increased by increasing the sintering pressure.

The average porosity of the sintered layers decreases by 2.25% when the holding time is increased from 3 to 5 min and by 0.91% when the holding time is increased from 5 to 7 min under the same pressure. The result demonstrates that the densification of the sintered layers increases and the rate of densification declines over holding time. Densification in the sintering process is accompanied by the elimination of crystal flaws, and the rate of densification slows as the concentration of crystal defects gradually decreases [25]. As a result, as the holding time increases, the amount of crystal flaws in the joint layer gradually reduces, and densification slows. When the porosity of the joint reduces to a certain degree, increasing the holding time has little influence on improving the quality of the sintered joint.

According to the above results, the large-area sintered joints obtained in this study with OVectDry as the drying method, sintering temperature of 250 °C, sintering pressure of 10–15 MPa, and holding time of 3–7 min have very close warpage and are free of delamination. Furthermore, increasing the sintering pressure has a greater impact on joint densification than increasing the holding time. Therefore, for reasons of performance and productivity, the recommended process condition is determined as follows: open-face convective drying, sintering temperature of 250 °C, sintering pressure of 15 MPa, and holding time of 5 min.

### 3.3. Thermal Fatigue Reliability Assessment of Large-Area Sintered Joints

**Figure 14:**
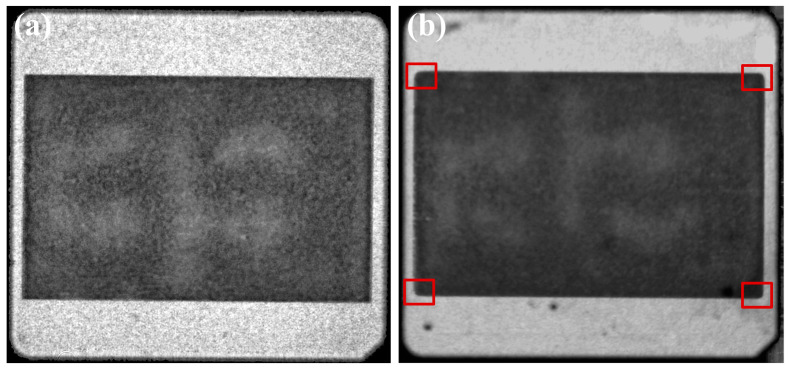
C-SAM images of the sintered joint after temperature cycling: (**a**) 0 cycles; (**b**) 1000 cycles.

**Figure 15:**
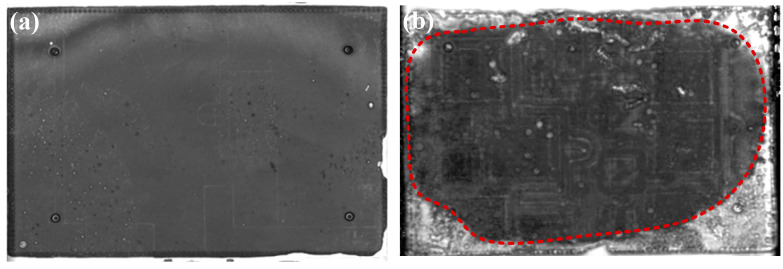
C-SAM images of the solder joint after temperature cycling: (**a**) 0 cycles; (**b**) 1000 cycles.

Based on the results of the process experiments, the recommended process condition was selected to prepare the samples for the thermal cycling reliability test. The interfacial delamination of the sintered layers was monitored using C-SAM, and the sintered joints are considered to have failed when the delamination area reached 20% of the initial joint area [26]. As shown in Figure 14, the C-SAM images show that the sintered layer did not cause significant delamination after 1000 cycles, and only slight delamination was observed at the edges and corners (areas labeled by red boxes). In order to demonstrate the reliability of sintered joints more sufficiently, SnSb5 solder joints with the same area of substrate bonding were prepared and subjected to the same thermal fatigue reliability test to compare with the sintered joints. As shown in Figure 15, the solder layer showed significant delamination after 1000 cycles (area outside the red dotted line), and the delamination area reached 21.32% of the initial joint area. The results of thermal cycling reliability tests showed that the thermal fatigue reliability of sintered joints was better than that of SnSb5 solder joints.

**Figure 16:**
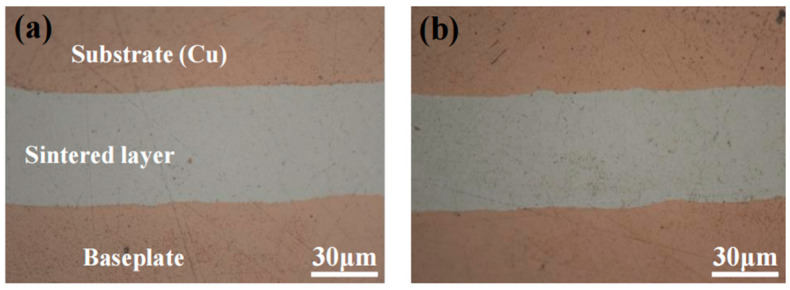
Cross-sectional microscope image of the sintered layer after temperature cycling: (**a**) 0 cycles; (**b**) 1000 cycles.

Figure 16 shows the cross-section light microscope images of the sintered layer of the large-area sintered joints initially and after 1000 temperature cycles. The images show that no cracks appear in the sintered layer of the reliability samples after 1000 cycles. The bond line thickness (BLT) of the sintered layer before and after temperature cycling was measured using the 3D profilometer. The average of the initial BLT is 49.32 μm, and the average of the BLT after 1000 cycles was 48.73 μm, which shows that the BLT of the sintered layers before and after the temperature cycling is very close.

The results of interfacial connection and BLT before and after temperature cycling demonstrate the outstanding thermal fatigue reliability of large-area sintered joints prepared under the recommended process conditions.

## 4. Conclusions

In this paper, the effects of different sintering processes (drying method, sintering pressure, and holding time) on the bonding quality of sintered joints were investigated, and the recommended sintering processes were obtained. The thermal fatigue reliability of sintered joints was also evaluated. The main conclusions obtained are as follows:

(1) Compared with CVectDry, OVectDry is more suitable for large-area sintering.
(2) The densification of the sintered layer increases with increasing sintering pressure and holding time, and the rate of densification reduces as holding time is extended. The recommended process condition is determined as follows: OVectDry, sintering temperature of 250 °C, sintering pressure of 15 MPa, and holding time of 5 min.
(3) After 1000 temperature cycles, the sintered layer is only slightly delaminated at the corners, with no cracks. The large-area sintered joints prepared under the recommended process condition have outstanding thermal fatigue reliability.

## Figures and Tables

**Table 1:** Process conditions of the samples for different drying methods.

Sample	Drying Methods	Drying Temperature/Time (°C/min)	Sintering Temperature/Pressure/Time (°C/MPa/min)
Sample 1	CVectDry	140/60	250/10/5
Sample 2	OVectDry		

**Table 2:** Process exploration conditions.

Sample	Drying Methods	Drying Temperature/Time (°C/min)	Sintering Temperature/Pressure/Time (°C/MPa/min)
Sample 3	OVectDry	140/60	250/10/3
Sample 4			250/10/5
Sample 5			250/10/7
Sample 6			250/15/3
Sample 7			250/15/5
Sample 8			250/15/7

## Data Availability

Data are contained within the article.
